# Roles of Chondroitin Sulfate Proteoglycans as Regulators of Skeletal Development

**DOI:** 10.3389/fcell.2022.745372

**Published:** 2022-04-08

**Authors:** Nancy B. Schwartz, Miriam S. Domowicz

**Affiliations:** ^1^ Department of Pediatrics, Biological Sciences Division, The University of Chicago, Chicago, IL, United States; ^2^ Department of Biochemistry and Molecular Biology, Biological Sciences Division, The University of Chicago, Chicago, IL, United States

**Keywords:** growth plate, signaling factors, chondrogenesis, degradation, regeneration, proteoglycans

## Abstract

The extracellular matrix (ECM) is critically important for most cellular processes including differentiation, morphogenesis, growth, survival and regeneration. The interplay between cells and the ECM often involves bidirectional signaling between ECM components and small molecules, i.e., growth factors, morphogens, hormones, etc., that regulate critical life processes. The ECM provides biochemical and contextual information by binding, storing, and releasing the bioactive signaling molecules, and/or mechanical information that signals from the cell membrane integrins through the cytoskeleton to the nucleus, thereby influencing cell phenotypes. Using these dynamic, reciprocal processes, cells can also remodel and reshape the ECM by degrading and re-assembling it, thereby sculpting their environments. In this review, we summarize the role of chondroitin sulfate proteoglycans as regulators of cell and tissue development using the skeletal growth plate model, with an emphasis on use of naturally occurring, or created mutants to decipher the role of proteoglycan components in signaling paradigms.

## Introduction

Proteoglycans, complex macromolecules that are prominent constituents of the ECM composed of a protein core to which are covalently attached variable length and composition glycosaminoglycan (GAG) chains (Schwartz, 2000). Because of their complex structure and chemistry, proteoglycans have been classified based on function, localization, and protein cores. To date, forty-three distinct proteoglycan-encoding genes have been identified, and are organized into four families based on their cellular and subcellular location, protein and genomic homologies, and unique protein modules shared by members of each specific family (Iozzo and Schaefer, 2015). Most proteoglycans interact with signaling molecules in multiple biological processes i.e., tissue development, wound healing and disease progression. These interactions are complex and often multivalent involving contributions by nonionic (hydrogen-bonding, Van der Waals and hydrophobic) forces, conformational changes, or clustering of binding complexes (Soares da Costa et al., 2017), or the ability of signaling factors to multimerize (Whalen et al., 2013). Among the families of sulfated proteoglycans, the heparan sulfate proteoglycans (HSPGs) are the best studied in terms of biointeractions with diverse ligands and various signaling molecules affecting cell behavior. A major ionic interaction is that between the carboxyl and sulfate groups in the GAG chains and positively charged amino acid (lysine and arginine) residues (Cardin-Weintraub sequence) in the N-terminal region of all hedgehog (HH) signaling molecules (Cardin and Weintraub, 1989) including sonic (SHH), indian (IHH) and desert (DHH). In addition to the HHs, proteoglycans with different modification patterns also function in fibroblast growth factor (FGF), wingless (WNT), transforming growth factor (TGFβ), chemokines and Slit/Robo signaling (Townley and Bulow, 2018).

## Chondroitin Sulfate Proteoglycans

In contrast to the HSPGs, the interactions of signaling molecules with chondroitin sulfate and dermatan sulfate proteoglycans (CSPG and DSPG), which are the focus of this review and are often the most abundant proteoglycans in tissues, are not as well understood. Chondroitin sulfate (CS) chains are found on multiple proteoglycans; the most common are the hyaluronan- and lectin-binding proteoglycans (hyalectans) which have structural similarity at both the protein and genomic levels. The hyalectan family consists of four members: aggrecan, versican, neurocan, and brevican, which all share a tri-domain structure: an N-terminal globular domain that binds hyaluronan, a central domain bearing the CS chains and a C-terminal region that binds lectins. The CS chains consist of repeating disaccharides of N-acetylgalactosamine (GalNAc) and glucuronic acid (GlcA) decorated with different degrees and patterns of sulfation on the disaccharides: GalNAc may have sulfate on C4 (CS-A), C6 (CS-C) or both C4 and C6 (CS-E), all catalyzed by specific sulfotransferases. The GlcA unit may also be sulfated on the C2 position (CS-B or CS-D) depending on where the sulfate residue is on GalNAc. Dermatan sulfate (DS) derives from chondroitin sulfate by inversion of GlcA to iduronic acid (IdA), catalyzed by an epimerase enzyme.

## Defining the Role of Proteoglycans in Signaling Pathways During Development

**FIGURE 1:**
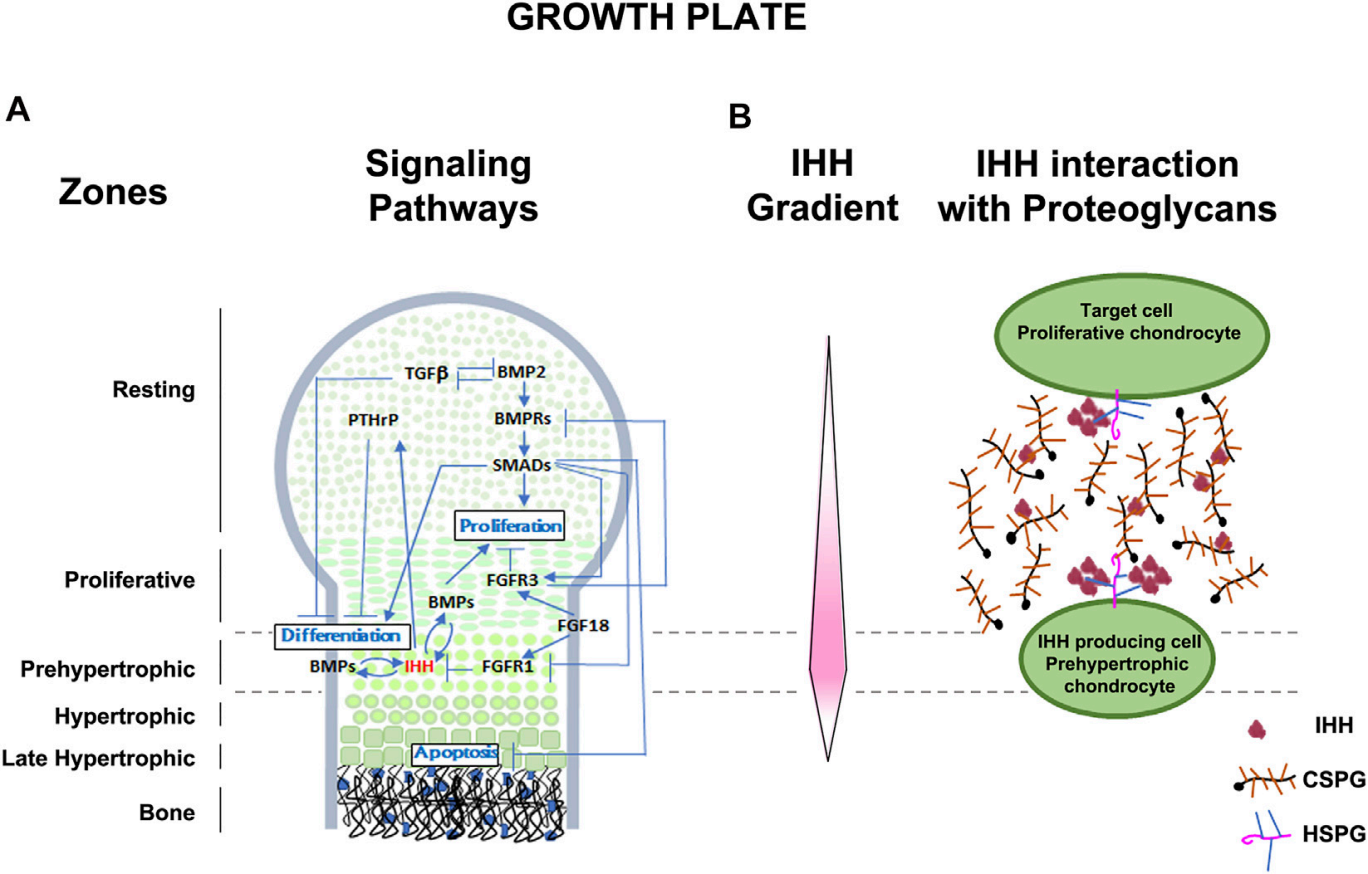
Schematic representation of the developing growth plate. **(A)** Zones of chondrocyte differentiation and known signaling pathways associated with chondrocyte proliferation, differentiation, and apoptosis are indicated. **(B)** Representation of the Indian hedgehog (IHH) gradient (protein concentration represented in pink intensity) and the interaction of IHH with extracellular CSPGs and plasma membrane associated HSPGs. IHH is secreted by prehypertrophic chondrocytes and the extended diffuse gradient expands to act on resting and proliferative chondrocytes. Extracellular matrix CSPGs help establish and maintain the extent of the diffusion gradient and protect IHH from degradation. Membrane associated HSPGs with higher affinity for IHH, act at the cell surface to bring IHH close to the plasma membrane for interaction with its receptors. Levels of multimerization of IHH is also dependent on lipoprotein particles not depicted here.

Determining direct relationships between proteoglycan structure/function and the bi-directional signaling that regulates development remains challenging, mainly because few tools exist that allow alteration or removal of specific GAG motifs or sulfate substitutions. However, some progress has been made using *in vitro* and *in vivo* approaches. Although general principles of CSPG interaction with signaling molecules have been shown in several developing tissue systems, we use as example the formation of skeletal structures with a focus on the growth plate of long bones, a transient cartilage template that is, replaced by bone. A complex and highly orchestrated program regulates growth plate cartilage morphogenesis in which chondroblasts proliferate, differentiate to chondrocytes, alter their shape, proliferate in stacks along the longitudinal axis, terminally differentiate to hypertrophic chondrocytes, and elaborate a mineralized vascularized matrix, which is then replaced by osteocytes (Karsenty and Wagner, 2002; Kozhemyakina et al., 2015). Multiple signaling pathways control the growth plate morphogenesis process including: IHH, FGF, TGFβ, bone morphogenic protein (BMP), parathyroid hormone-related peptide (PTHrP), SMAD6 and SMURF, all of which play unique and sometimes interacting roles in growth plate morphogenesis (Wang et al., 2021) (Figure 1A).

**FIGURE 2:**
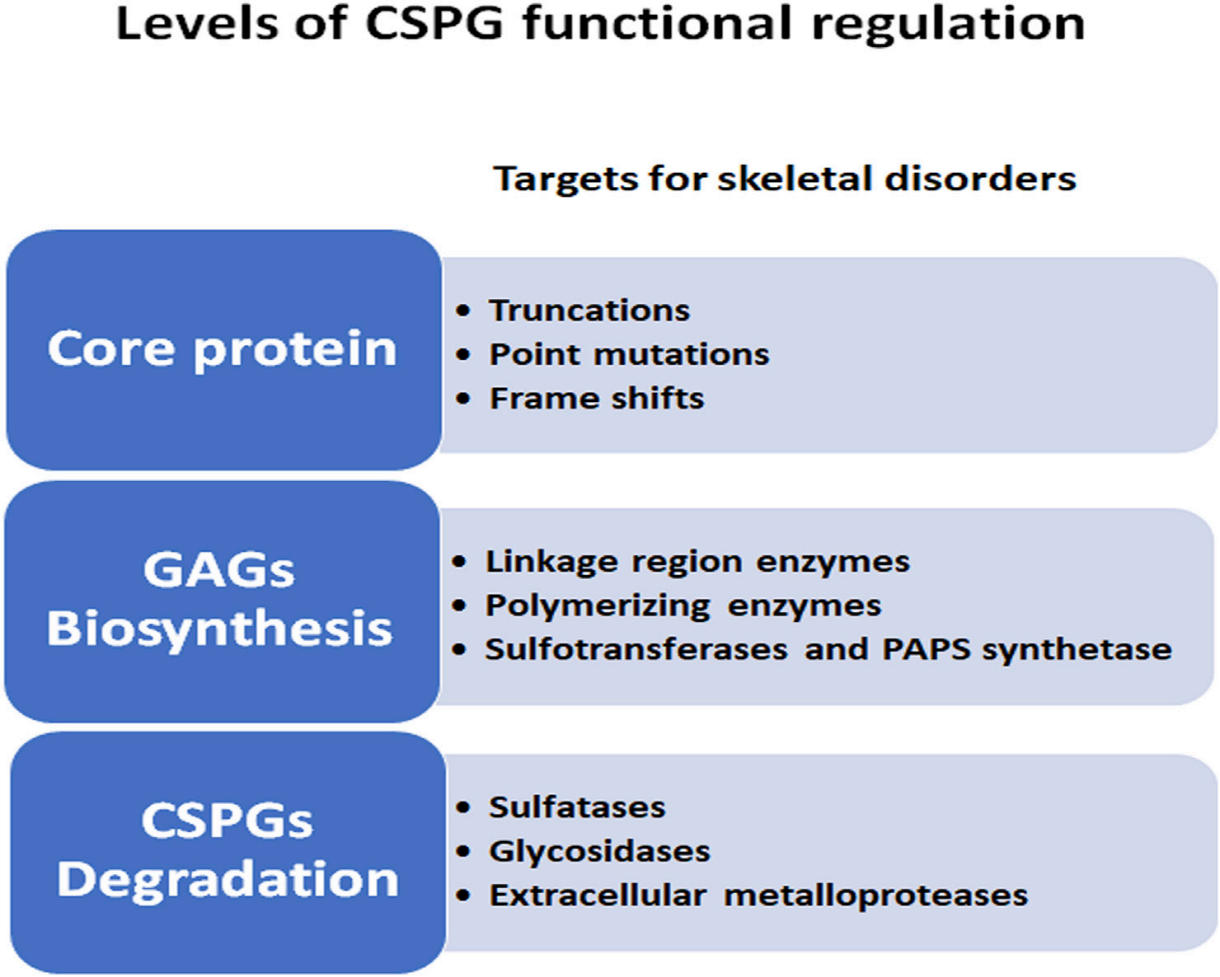
Levels of CSPG functional regulations during skeletal development. CSPG complexity is illustrated by the multiple levels at which the synthesis and degradation of these molecules can affect the outcome of normal growth plate development.

Some well documented examples of the mechanisms by which signaling pathways are necessary for cartilage morphogenesis are illustrated in Figure 1A and include the IHH-PTHrP negative feedback loop which regulates the size of the proliferative zone and onset of hypertrophy (Vortkamp, 2001). PTHrP, secreted by the resting zone, preserves the reservoir of progenitor cells and promotes chondrocyte proliferation by interacting with IHH secreted by hypertrophic chondrocytes (Mizuhashi et al., 2018). Conversely, IHH antagonizes PTHrP signaling and promotes chondrocyte hypertrophy in the lower segment of the growth plate (Lee et al., 2019). Members of the TGFβ family promote chondrogenesis in undifferentiated mesenchyme cultures (Karsenty and Wagner, 2002), while long bone chondrocyte proliferation and hypertrophy is inhibited by TGFβ (Serra and Chang, 2003). Targeted deletion of the TGFβ2 gene product alters the overall size and shape of limb rudiments (Sanford et al., 1997), and naturally occurring mutations in the TGFβ2 gene cause Camurati-Engelmann Disease, characterized by thickening of the long bone collar (Campos-Xavier et al., 2001). Another major signaling family, the BMPs, positively regulate both chondrocyte proliferation and hypertrophy (Horiki et al., 2004), as shown in mice with mutations in the BMP receptor type 1B that develop brachyactyly (Baur et al., 2000), and in mice which over-express SMAD and SMURF (negative regulators of BMP signaling), leading to chondrocyte hypertrophy and dwarfism (Horiki et al., 2004). The patterning of bone and joints also requires the interaction of multiple signaling pathways, including BMP members, HH, WNT, and FGF families (Archer et al., 2003; Baldridge et al., 2010). Thus, as these examples illustrate, the processes of cartilage, bone, and joint development is dependent on multiple morphogens, growth factors, and cytokines. However, understanding the role of CSPGs, the major proteoglycans in the growth plate, in influencing the functions of these signaling molecules and pathways during skeletal development remains limited. In this review we provide evidence of the roles by which each major feature (core protein, GAG chains, sulfation) of proteoglycans contribute to regulation of chondrogenesis (Figure 2).

## Role of CSPG Core Protein

Several studies have described critical roles for CSPGs such as aggrecan, which is broadly expressed by chondrocytes, and versican, which is transiently expressed in undifferentiated mesenchyme, in chondrogenesis and joint morphogenesis (Schwartz and Domowicz, 2002; Snow et al., 2005; Shepard et al., 2007; Domowicz et al., 2009; Choocheep et al., 2010; Lauing et al., 2014). For most of these studies, mutant models of proteoglycan biosynthesis and metabolism have helped to unravel the role of proteoglycans in skeletal formation and maintenance. In fact, the strongest evidence that CSPGs are essential during differentiation of chondrocytes and maintenance of skeletal elements rests on the demonstration of abnormalities in CSPGs concomitant with aberrant growth patterns in human and animal models (Melvin and Schwartz, 1988; Schwartz and Domowicz, 1998). In particular, mutations in the aggrecan gene are the cause of several chondrodysplasias and inherited skeletal disorders in humans and animals (Schwartz and Domowicz, 2002; Schwartz and Domowicz, 2014). Mutations in the aggrecan core protein gene have been identified in human skeletal disorders including: spondyloepimetaphyseal dysplasia with premature and severe osteoarthritis and osteochondritis (Gleghorn et al., 2005) and the recessive skeletal dysplasia EMD aggrecan-type which results from a missence mutation affecting the C-type lectin domain of aggrecan (Tompson et al., 2009; Stattin et al., 2010). To date, eight human genetic diseases involving defects in aggrecan, now coined the aggrecanopathies (Gibson and Briggs, 2016) have been identified, but the impact of these aggrecan defects on signaling in humans has not yet been fully explored.

One of the earliest studied animal models was nanomelia (*nm*), a lethal chondrodystrophy of fowl (Landauer, 1965). The *nanomelic* chick cartilage can synthesize CS chains, but aggrecan core protein is absent due to a single nucleotide change that results in a premature stop codon in the aggrecan gene (Argraves et al., 1981; Li et al., 1993; Schwartz et al., 1993; Vertel et al., 1994). This severely truncated core protein is not glycosylated or transported through the secretory pathway leading to an altered cytoarchitecture (densely packed cellular growth plate devoid of matrix) and homeostasis (increased proliferation of hypertrophic chondrocytes and increased cell death in the proliferative zone) (Domowicz et al., 2000). Since all of these phenotypes are regulated by signaling pathways, these aggrecan mutants present ideal models for investigating the core protein interactions with growth plate regulators. Thus, further studies documented that loss of aggrecan results in defects in morphogen gradient distribution and gene expression profiles of the critical chondrocyte regulators (IHH, BMP, and FGF) (Domowicz et al., 2009; Schwartz and Domowicz, 2014).

**FIGURE 3:**
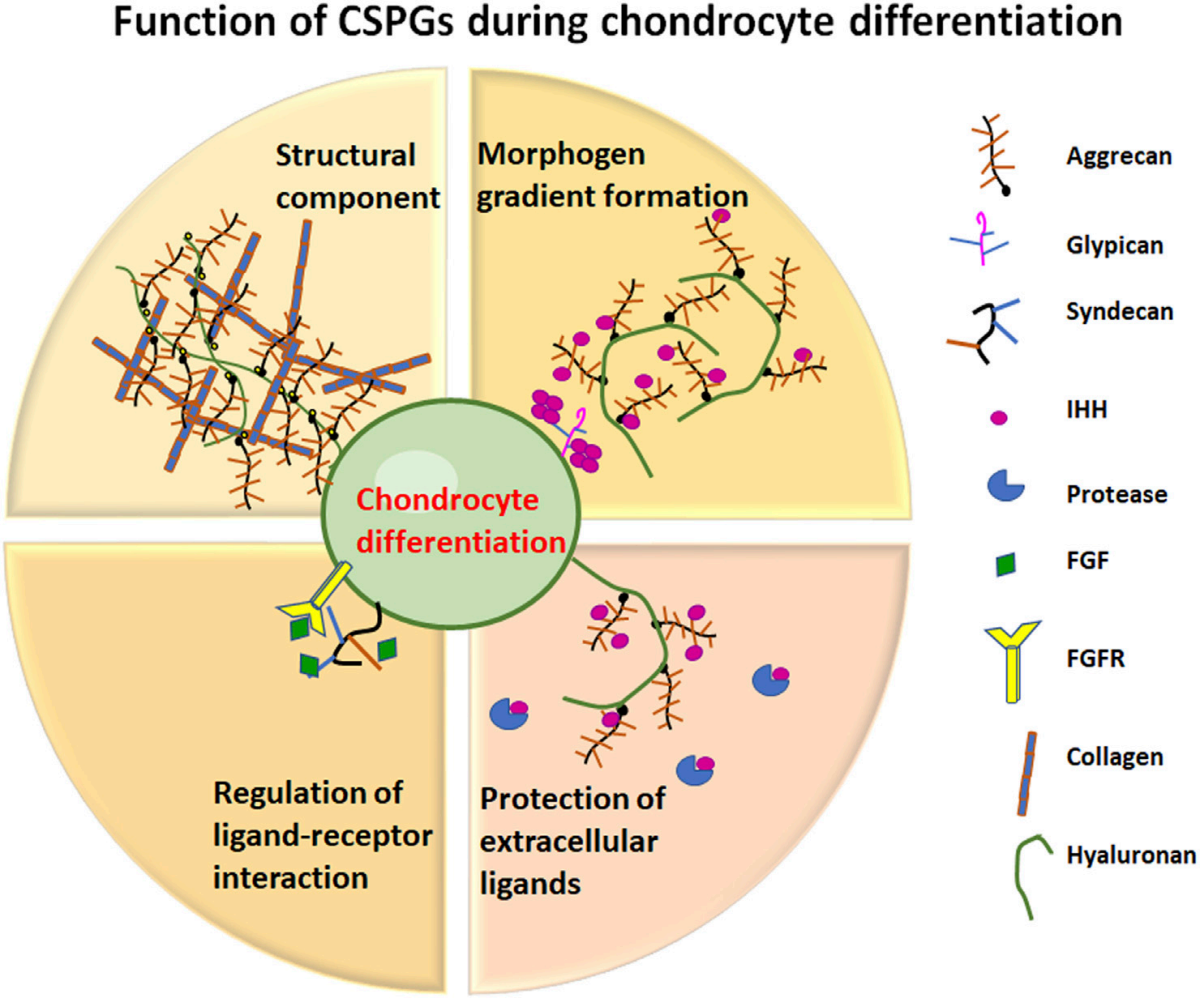
Function of CSPGs during chondrocyte differentiation. Representation of the different roles that CSPGs play in cartilage development.

A similar lethal mutation in the aggrecan genes of the cartilage-matrix deficiency (*cmd*) mouse is due to a 7-bp deletion in exon 5, resulting in a premature stop codon and no aggrecan product (Watanabe et al., 1994). A second mutation within the same locus and generating a similar phenotype, *cmd* ^*bc*^, has been identified as the complete loss of exon 2 to 18, resulting in a significantly shortened mRNA and production of no aggrecan core protein (Krueger et al., 1999). In a landmark study, a novel transgenic mouse line (Tg COL2A1-ACAN) expressing a chick ACAN coding sequence driven by the mouse *Col2A1* promoter has enabled the generation of cmdbc/cmdbc; Tg (COL2A1-ACAN) rescue embryos (Lauing et al., 2014). Robust re-expression of aggrecan in rescue embryos reversed the defects in different skeletal elements to varying degrees, most notably the reappearance of a hypertrophic zone and production of *Col2a1* and *Col10a1* in the limb growth plate. As well, transgene expression in rescue mice restored: i) Sox9 expression in resting and proliferative zones similar to wild type; ii) an increase in *Ihh* mRNA production in more chondrocytes in the pre-hypertrophic region; iii) relatively normal expression of *Ptch1*, the receptor of IHH within the bone marrow near the chondro-osteo junction, perichondrium and proliferative zones similar to wild type; iv) strong re-expression of *Fgfr3*, which encodes the receptor for negative regulators of chondrocyte proliferation such as FGF9 and FGF18 in the proliferative and early hypertrophic zones of the growth plate; all these features closely resemble those found in wild-type embryos. Taken together, the data obtained from RT-PCR, immunochemistry and mRNA *in situ* analyses confirm that the presence of aggrecan in the growth plate ECM is fundamental to maintaining normal expression and spatial localization of the essential signaling molecules that regulate chondrocyte organization, morphology, and maintenance during growth plate development (Lauing et al., 2014) (Figure 3).

## Role of GAG Chain Synthesis

These cited examples clearly present a requirement for CSPGs as demonstrated by mutations in the core protein, which lead to reduced or total absence of CSPGs. However, the complexity of CS/DSPGs and potential interplay with signaling molecules may also be due to interactions involving the long, linear GAG chains of repeating disaccharide units, as well as the contribution of sulfation components of proteoglycans. GAG chain initiation for CS, DS, HS, and heparin begin with addition of Xyl to a serine hydroxyl embedded in a specific core protein peptide sequence (Bourdon et al., 1987; Krueger et al., 1990), catalyzed by the chain-initiating enzyme xylosytransferase (Kearns et al., 1993; Schwartz, 1995). CS/DSPG GAG chain synthesis continues with addition of two galactose (Gal) residues and a GlcA residue, catalyzed by unique-glycosyltransferases (Schwartz and Rodén, 1974; Schwartz and Roden, 1975; Schwartz, 1976). In contrast to the common linkage region structures shared by most proteoglycans, their structural diversity and functional complexity derives from the long linear unbranched GAG chains comprised of unique repeating disaccharide units, which are then substituted with O- or N- linked sulfate groups. GAG chains engage in regulation of biological processes by interacting with various ligands; however, very little is known about these interactions since structural analysis of GAGs is difficult (Song et al., 2021).

### Xylosylation-Initiation of CS Chains

The first step of GAG chains initiation is catalyzed by one of two paralogs XYLT1 AND XYLT2 (Voglmeir et al., 2007). Interestingly, five homozygous *XYLT1* mutations were reported in individuals with Desbuquois dysplasia (DBQD) type 2, leading to severe chondrodysplasias which suggests a requirement for xylosyltransferase during skeletal development (Bui et al., 2014). Sulfate labeling of fibroblast from patients with certain *XYLT1* mutations show predominant sensitivity to CS digestion, suggesting synthesis of CSPGs are affected by partial loss-of-function *XYLT1* (Bui et al., 2014). Another short stature syndrome caused by a homozygous mutation in *XYLT1* exhibited a potential localization defect since the enzyme was diffusely distributed throughout the cytoplasm (Schreml et al., 2014) rather than in the ER and early Golgi (Hoffmann et al., 1984; Vertel et al., 1993). A third type of skeletal disorder, Baratela-Scott syndrome (Baratela et al., 2012) is caused by homozygous mutations in *XYLT1* due to hypermethylation defects (LaCroix et al., 2019). Homozygous mutations in *XYLT2* cause spondyloocular syndrome, which exhibits skeletal defects, as well as ocular, cardiac, auditory system defects, and learning difficulties in patients with this disorder (Munns et al., 2015; Taylan et al., 2016; Taylan et al., 2017; Umair et al., 2018). The different clinical manifestations of *XYLT1* and *XYLT2* disorders, suggest potential functional distinctions between the two enzymes including: use of distinct core protein substrates, differential spatiotemporal expression, and inability to compensate for each other (Mizumoto and Yamada, 2021). These fascinating issues, as well as how signaling pathways are affected as a consequence of these *XYLT* mutations that lead to the devastating phenotypes, remain unexplored in these human hereditary disorders.

A better understanding of the role of xyloxyltransferases has come from the study of an animal model with a xylosyltransferase mutation. The phenotype of the “*pug*” mouse has a missense mutation in *Xylt1*, resulting in skeletal abnormalities (Mis et al., 2014). This recessive dwarf mouse mutant (*pug*) was identified from an N-ethyl-N-nitrosourea (ENU) mutagenesis screen, and exhibits reduced skeletal element lengths, normal growth plate patterning and no change in chondrocyte proliferation; however, *pug* mutants display premature maturation and early ossification leading to the disproportionate dwarfism. The mutation in *Xylt1* disrupts enzyme activity and leads to reduction in the number of GAG chains in *pug* mutant proteoglycans. Furthermore, XYTL1 was mislocalized as it was not observed in the cis-Golgi as previously shown in wild type mice (Kearns et al., 1993; Vertel et al., 1993; Muller et al., 2006; Schön et al., 2006). Thus, this model provides a valuable resource for studying the impact of lack of CS-chains on signaling in growth plate development (Mis et al., 2014). As might be expected, decreased XYLT1 activity in *pug* mutants leads to complex signaling defects, since the *Xylt1* mutation affects synthesis of both HSPGs and CSPGs (shown by sulfate labeling). Furthermore, the phenotype described above (delayed chondrocyte maturation) suggests IHH and FGF signaling pathways may be affected. As predicted, an increase in *Fgfr3* levels, but no downstream changes in MAPK signaling were observed. Concomitantly, an up-regulation of short-range IHH signaling was observed, while long-range IHH signaling through PTHrP, which inhibits chondrocyte maturation, was not affected. These results suggest that the premature maturation of *pug* chondrocytes may not be influenced by changes in IHH and FGF signaling. Rather proteoglycans, in addition to regulating diffusion of key signaling molecules, may provide maturation cues to chondrocytes independent of IHH and FGF signaling. Furthermore, since the *Xylt1* mutation affects both CSPG and HSPG production, the IHH responses also suggest that these two proteoglycans may function differently in regulating diffusion of IHH through the ECM, with CSPGs expanding the IHH diffusion domain and HSPGs restricting the domain. The premature-maturation *pug* phenotype, in the absence of signaling changes, suggests a novel proteoglycan cue that influences timing of chondrocyte maturation (Mis et al., 2014). Although the *pug* mutant mouse shares characteristics with a human dwarfism (Azouz et al., 1998), the skeletal defects appear more severe in the mouse mutant and thus may serve as a potential candidate for as yet unidentified gene defects underlying short stature or dwarfism phenotypes in humans. Most importantly, understanding how signaling pathways are affected in this mutant may lead to potential new therapeutic targets.

### Synthesis of CS, DS, and HS Linkage Region

In addition to the critical importance of xylosyltransferases to initiating the tetrasaccharide linkage region (GlcA-Gal-Gal-Xyl-o-) of CS, DS, and HS, mutations in the other three enzymes also cause hereditary diseases. Compound heterozygous and homozygous mutations in *B4GALT7*, the enzyme that adds the first Gal residue to -serine-o-xyl- cause Ehlers-Danlos syndrome (EDS) spondylodysplastic type 1 (Malfait et al., 2017; Malfait et al., 2020) which is characterized by short stature, muscle hypotonia and bowing of limbs. Because of multiple mutations in the same gene causing differential substrate selectivity and/or intracellular location, patients may exhibit defects in CS, DS, and even HS GAG chains leading to a wide range of symptoms (Salter et al., 2016; Ritelli et al., 2017; Mihalic Mosher et al., 2019). Larsen syndrome of Reunion Island Syndrome is caused by a homozygous mutation in B4GALT7 and exhibits a clinical spectrum that overlaps with EDS spondylodysplastic (Cartault et al., 2015). Compound heterozygous mutations in B3GALT6 encoding GALT-II which transfers the second Gal residue to the growing linkage region, leads to two disorders: Ehlers-Danlos syndrome spondylodysplastic type 2 (Malfait et al., 2013; Nakajima et al., 2013) which produces less CS, DS, and HS, as well as spondyloepimetaphyseal dysplasia (Nakajima et al., 2013), which predominantely reduces HSPG3 (perlecan), while CS- and DSPGs (versican and decorin) levels are normal (Ritelli et al., 2015). Lastly, multiple homozygous or heterozygous mutations, have thus far been identified in the last linkage region enzyme, B3GAT3 that encodes the glucuronosyltransferase which adds a GlcA residue to the Gal-Gal-Xyl- backbone (Budde et al., 2015; Alazami et al., 2016; Byrne et al., 2020). Again, multiple mutations in the same gene affect synthesis of CS, DS, and HS to varying degrees and lead to syndromes with a broad spectrum of phenotypes (Larsen-like syndrome B3GAT3 type, Spondyloepipheseal dysplasia with congenital joint dislocation and Pseudodiastrophic dysplasia). Unfortunately, no information is yet available on identifying affected signaling pathways, which are required to understand the underlying pathogenic mechanisms.

### GAG Chain Elongation

Following synthesis of the CSPG linkage region, the linear repeating disaccharide units of GalNAC and GlcUA are added, catalyzed by glycosyltransferases that work in concert with sulfotransferases to accomplish polymer elongation and sulfation (Silbert and Sugumaran, 1995; Schwartz, 2000; Schwartz, 2014). Chondroitin sulfate synthase1 (CHSY1) is one of six glycosyltransferases involved in CS chain elongation (Kitagawa et al., 2001). Although all six enzymes are localized to the site of CSPG synthesis only CHSY1, CHSY2 and chondroitin sulfate glucuronyltransferase (CHPF2) catalyze addition of GalNAC and GlcUA saccharide units to elongate CS and DS. These enzymes are co-expressed with aggrecan in the pre-hypertrophic zone of embryonic growth plates (Sakai et al., 2007). Loss of function mutations at the Chsy1 locus have been identified in human Syndromic recessive preaxial brachydactyly (Li et al., 2010; Tian et al., 2010). Mouse mutants for the three CS/DS elongation enzymes have been generated; only *Chsy1*−/− exhibited brachypodism with a patterning defect in distal phalangeal elements, achondrodysplasia and decreased bone density, caused by a reduction in CS chains and a shift in cell orientation. Transcriptome analyses of candidate genes implicated in joint formation, as well as *in vivo* analysis of growth factor signaling by FGF, TGFβ, BMP, WNT3A, NOTCH and HH in mouse embryonic fibroblasts (MEPs) and primary chondrocytes, suggested that IHH distribution was altered and that mutant MEPs are more sensitive to HH stimulation. Together these findings suggest that IHH signaling is disrupted, but differences in signaling may be secondary to changes in chondrocyte orientation (Wilson et al., 2012).

## Role of Sulfation

Lastly, the impact of sulfation molecular diversity and patterning of CS/DS chains on signaling have also been investigated. Sulfation is particularly influential in GAG cross talk either indirectly by regulating protein folding via steric hindrance, exclusion or recruitment, or directly through electrostatic interactions that often are sequence specific. In the skeleton, sulfation plays two main roles: to generate osmotic swelling pressure which enables cartilage to withstand compressive loads and to foster direct cell-proteoglycan interactions with specific growth factors or signaling molecules. Interestingly, sulfation patterns change with maturation of cartilage (Bayliss et al., 1999) with an increasing ratio of CS-6 to CS-4 sulfated GAG chains and a diminished sensitivity to TGFβ (Hickery et al., 2003). Similarly, changes in sulfation patterns greatly influence skeletal development and maintenance by altering interactions with systemic soluble factors (IHH, PTH, FGFs, TGFβ and BMPs) (Klüppel et al., 2005), verifying that imbalance in GAG sulfation can modify the functioning of these signaling pathways. As mentioned, several sulfotransferases are involved in the 4- and 6- sulfation of GalNAC units and the GlcA unit. Examples of altered GAG sulfation involving sulfotransferases that cause abnormalities have been identified for both: mutations in chondroitin-6-sulfotransferase-1 (C6ST-1) which are associated with chondrodysplasia and progressive spinal involvement (Thiele et al., 2004), while mice deficient in chondroitin-4-sulfotransferase (C4ST-1) exhibit a more severe chrondrodysplasia. Detailed analysis of the mutant growth plate showed abnormal CS localization, chondrocyte differentiation and orientation, and strong up-regulation of TGFβ signaling with concomitant down-regulation of BMP signaling (Klüppel et al., 2002; Klüppel et al., 2005).

In addition to sulfotransferase-caused signaling defects, limiting the sulfate substrate for the sulfotransferases, phosphoadenosine phosphosulfate (PAPS), also leads to chondrodystrophies in mice (Orkin et al., 1976; Schwartz et al., 1978; Kurima et al., 1998) and humans (Faiyaz ul Haque et al., 1998). The brachymorphic (*bm*) mouse model (Sugahara and Schwartz, 1979; Sugahara and Schwartz, 1982a; Sugahara and Schwartz, 1982b; Sugahara and Schwartz, 1982c) has a mutation in the PAPSS2 gene which encodes PAPS synthetase 2 (PAPSS2), one of two isoforms in mammals that catalyze the synthesis of the universal sulfate donor (PAPS) (Kurima et al., 1998). At birth, mice are normal size but as development proceeds *bm* mice exhibit a 50% reduction in limb length, 25% reduction in axial skeleton and a normally organized growth plate but with a reduction in all zones (Schwartz et al., 1978). Aggrecan (*Acan*) and *Col10a1* mRNA expression were comparable in wild type and *bm* mutants. In contrast, using a set of antibodies with specificity for all functional sulfate epitopes, immunohistochemistry revealed reduction in CS-4 and CS-6 epitopes and an increase in the CS-0 epitope in the *bm* growth plate EMC, compared to wild type. In contrast, N-sulfated HS showed comparable staining in wild type and *bm* growth plate. These data were verified by FACE and ^35^S-sulfate incorporation experiments; only a reduction of sulfate incorporation into CSPGs of the predominantly CS-4 species and no change in HS-sulfate content in *bm* cartilage was observed; establishing the *bm* mouse as an excellent model for investigating the role of under-sulfated CS interactions with signaling molecules during cartilage development (Cortes et al., 2009).

Analysis of growth plate signaling showed that the PTHrP receptor (*Pthr1*) was expressed at high levels in the pre-hypertrophic zone in both *bm* and wild type. In contrast, *Fgfr3* and *Ihh* (expressed in the pre-hypertrophic zone) and its receptor patched (*Ptch1*) expressed in the proliferative zone exhibited decreases in mRNA levels in *bm* cartilage by three methods, mRNA *in situ*, RT-PCR, and immunohistochemistry. In particular, IHH protein was not uniformly distributed between chondrocytes in *bm* samples, rather a restricted diffusion pattern characterized by protein aggregation was observed. The abnormal IHH distribution was verified by crossing *bm* mice with LacZ *Ptch* ± mice and determining the ratio of Gli activator (*Gli1*/*Gli2*) to Gli repressor (*Gli3*) to measure IHH pathway activation (Hilton et al., 2005). Since, a major function of IHH is to regulate chondrocyte proliferation, cell division was assessed. Significant decreases in BrdU-incorporation were observed, especially in the distal proliferative zone which correlates with the region of restricted IHH diffusion and decreased PTCH1 activation, verifying a decrease in cell division due to a disruption in IHH signaling in the under-sulfated *bm* growth plate (Cortes et al., 2009).

As with most previous studies on reciprocal interactions between signaling factors and CSPGs, the results are compelling, but not biochemically definitive. This ultimate goal was accomplished by three direct approaches. First, quantitative binding curves between IHH-alkaline phosphatase (AP) fusion protein and HS and CS GAG chains (with CS-4, CS-6, and unsulfated CS-0 motifs) showed a gradient of binding affinity (Kd) and binding capacity (Bmax) in order: HS, CS-4, CS-6, CS-0. Since CS-4 is the predominant species in postnatal cartilage and the binding affinity and capacity is higher for the CS-4 to CS-0 motif, a reduction in CS-4 is commensurate with abnormal IHH signaling. Secondly, to demonstrate that IHH interacts with CS specifically and does so through the IHH N-terminal Cardin-Weintraub motif, this motif was mutated which resulted in complete loss of binding to both HS and CS chains, suggesting that the interaction between IHH and CS is primarily mediated through this motif. Lastly, a direct interaction between CSPG and IHH-AP was demonstrated by quantitative immunoprecipitation with a specific aggrecan antibody, demonstrating that the major cartilage CSPG, aggrecan, directly interacts with IHH. Taken together, the biochemical and genetic evidence suggest a biological mechanism whereby undersulfated CSPGs result in restricted IHH diffusion through the ECM leading to a reduction in chondrocyte proliferation, which significantly impacts skeletal growth in the *bm* mutant (Cortes et al., 2009).

## Summary of CSPG Structural Components in Regulation of Its Synthesis

In addition to providing the most definitive evidence to date that molecular interactions occur between signaling factors and CSPGs, these landmark studies extend our understanding of the biological consequences of these interactions. First, the severe-to-mild spectrum of chondrodystrophies observed in CSPG-deficient models correlates directly with the location of the mutations in the CSPG synthetic pathway. Absence of aggrecan core protein (*nanomelic* chick and *cmd* mouse) leads to lethal phenotypes (Li et al., 1993; Krueger et al., 1999; Schwartz and Domowicz, 2002), whereas GAG chain addition (*Pug*) or sulfation (*bm*) models present with milder chondrodyplasia phenotypes (Cortes et al., 2009; Wilson et al., 2012; Lauing et al., 2014; Mis et al., 2014). Secondly, these studies provide a rational for the observations in complex ECMs consisting of more than one proteoglycan. In the cartilage matrix, CSPG is the predominant proteoglycan and contains a large number of CS chains per core protein (Krueger et al., 1990), therefore requiring more PAPS to sulfate the CS chains. In contrast, HSPGs contain fewer HS chains to be sulfated (Knox and Whitelock, 2006). However, HS sulfotransferases have higher affinity for PAPS and therefore result in preferential sulfation of HS chains even if PAPSS2 levels are reduced. Furthermore, the *bm* phenotype, in which only CSPG sulfation is reduced, is opposite of HS synthesis mutants. In particular, in the *Ext1* gene trap mutant, reduction in HS results in an increased range of HH signaling (marked by increases in *Ptch1* and *Pthrp* mRNA), increased chondrocyte proliferation and expansion of the proliferative zone (Koziel et al., 2004). In another study, additional HH binding sites were found using structural approaches (Whalen et al., 2013) that allowed multimerization of HSPGs close to the cell membrane needed for interaction with its receptor, while CSPGs, that are more broadly distributed in the ECM and have a lower affinity for HH, establish formation of the HH gradients, which may expand several cell-lengths from the site of production (Cortes et al., 2009) (Figure 1B). Thus, both CSPGs and HSPGs function as IHH modulators, and in concert, influence long-range HH signaling in the ECM growth plate. Third, it is important to highlight that temporal changes in GAG levels or composition are also critical to the mechanistic consequences in growth plate development. For example, reduction in GAGs during the formation of the growth plate (i.e., *nm*, *cmd*, *pug*) leads to accelerated maturation of chondrocytes, while reduction in GAGs in the mature growth plate (i.e., *bm*) leads to changes in morphogen distribution and altered rate of cell division. Lastly, two other mutant mouse models, a Golgi PAP phosphatase (Frederick et al., 2008) and nucleotidase *Jaws/Bpnt2* (Sohaskey et al., 2008) both show only under-sulfated CSPGs and severe chondrodysphasias. All these findings suggest that sulfated CSPGs function in IHH signaling processes independent of HSPGs, and illustrate how bidirectional communication processes are especially important for regulating cell differentiation during normal tissue development (Gjorevski and Nelson, 2009; Clause and Barker, 2013).

## Signaling in CSPG Degradation

As just summarized, many of the major developmental signaling pathways acting on growth plate cell populations function directly and indirectly through CSPGs, which also reciprocally influence the activity of these signaling pathways. As well, defects in CSPG-GAG metabolism has the potential to disrupt the function of essential regulators and is likely a major underlying mechanism for abnormal skeletogenesis progression (Alliston, 2010).

### Mucopolysaccharidoses

The mucopolysaccharide (MPS) disorders exhibit tissue-specificity for GAG metabolism and function. Those MPSs involving lysosomal storage of CS/DSPG and KSPG families are associated with skeletal disorders (MPS VI, MPS IVA, MPS VII). In contrast, HSPGs are usually associated with central nervous system (CNS) pathology (MPS III), and MPS enzymes common to multiple GAG pathways cause both skeletal and CNS pathology (e.g., MPS I and II). An elegant example of the critical role of CSPG synthetic and catabolic enzymes in growth regulation are the consequences of either deletion of C4ST or MPS VI, both of which lead to skeletal malformations. Although MPS VI and C4ST phenotypes are not identical, there are similarities indicating that both synthesis and degradation of CSPGs cause cellular de-regulation of growth plate development. As discussed earlier, C4ST deficiency hyper-activates TGFβ signaling while down-regulating BMP signaling (Klüppel et al., 2005). MPS VI is due to N-acetylgalactosamine-4-sulfatase deficiency and contributes to degradation of CS-4 and DS, leading to severe skeletal disorders in humans (Litjens and Hopwood, 2001). Since CS-4 is the major GAG in the cartilage growth plate, a mechanistic link between CS-4 and MPS-VI bone shortening and growth plate disorganization is proposed (Alliston, 2010). Although the expression of TGFß and other TGFß-regulated genes are disrupted in both chondroitin-4-sulfotransferase 1 (C4ST-1 also known as CHST11) (Klüppel et al., 2005) and MPS-VI (Simonaro et al., 2005), detailed aspects of the mechanisms controlling disruptions in skeletogenesis in the mucopolysaccharidoses largely remain to be determined.

### Osteoarthritis

Understanding functional interactions between components of the ECM and signaling pathways that control synthesis of the ECM components is also critical to degrative diseases like osteoarthritis (OA), a common degenerative disorder with no current disease-modifying therapies. This skeletal disorder is due to degradation of the major CSPG, aggrecan, mostly by specific enzymes, aggrecanases (ADAMTS-5). These aggrecan degradative enzymes are upregulated by mediators associated with joint inflammation or tissue overloading (Roughley and Mort, 2014). Interestingly, deletion of the TGFβ receptor type II gene, a component of the TGFβ/SMAD3 signaling system which represses chondrocyte hypertrophic differentiation required for maintaining articular cartilage (Yang et al., 2001), leads to a progressive osteoarthritis-like phenotype in mice (Shen et al., 2013), again illustrating disease causation by both CSPG alteration and major signaling pathways. As well, the number of mutations in the TGFβ1 signaling cascade with increased OA risk, provide strong evidence of a protective role for TGFβ. Thus, clinical trials to assess treatment of OA by intra-articular injection of allogenic chondrocytes transduced to express TGFβ1 have shown improved range of movement and reduced pain scores (Ha et al., 2012) and are being continued to assess long term improvement (Lee et al., 2020).

Other approaches have also been attempted to improve the OA pathology: inhibiting the degradative enzymes (Glasson et al., 2005) or increasing the repair capacity of cartilage through delivery of factors that promote ECM synthesis; although challenging the latter approach has been more rigorously investigated [reviewed in (Patel and Lim, 2019)]. To test whether the osteoarthritis degenerative process may be retarded by enhancing production of aggrecan, individual or combinations of growth factors including: FGF2, TGFβ, and members of the BMP family, have been delivered via gene therapy to osteoarthritis models (Trippel et al., 2007; Shi et al., 2013). More recently, direct intra-articular injection of autologous plasma containing high platelet levels, that are activated by cartilage ECM proteins, thereby releasing their anabolic growth factors (TGFβ1, PDGF, IGF, FGF2) and promoting aggrecan synthesis (Everts et al., 2020) have also been used as therapy. While numerous *in vitro* studies and clinical trials with plasma have produced mixed results (McClurg et al., 2021), intra-articular injection of individual anabolic factors still remains a particularly active area. As example, FGF18 significantly reduced cartilage degeneration in a rat OA model (Moore et al., 2005; Mori et al., 2014). Pharmaceutical companies have produced a modified form of FGF18 (i.e., sprifermin) that stimulates proliferation of chondrocytes, increases GAG production and decreases ADAMTS5 expression (Gigout et al., 2017). Human clinical trials using cartilage structural parameters and patient-reported pain and stiffness scores as outcomes, showed some improvements in cartilage thickness, but no change in function or pain scores (Hochberg et al., 2019). Lastly, WNT signaling promotes hypertrophic differentiation of chondrocytes with deleterious effects on cartilage homeostasis (Usami et al., 2016; Dell'Accio and Cailotto, 2018); thus, inhibition of WNT signaling is also being explored for OA therapy. Several small molecule inhibitors have been developed and are in clinical trials, with promising results (Deshmukh et al., 2019; Yazici et al., 2020). As well, introduction of genetically engineered cells (TissueGene-C) over-expressing TGFβ packaged into a cell line (Lim et al., 2017) has entered Phase 3 Clinical trials (Mobasheri et al., 2020). Clearly, stimulating chondrogenic differentiation with known growth factor genes/proteins is a potential strategy for *ex vivo* gene therapy modalities in the complex cartilage ECM (Uzieliene et al., 2021). Furthermore, on the basis of the lessons learned from the skeletal developmental models, inhibiting chondrocyte maturation and maintaining high levels of CSPG biosynthesis are critical to harnessing the potential of these novel therapies.

## Signaling in Regeneration

Because of the complexity of the ECM with multiple physical, biological, and chemical interactions involving temporal control of signaling molecule networks, it is challenging to recreate these environments experimentally. As well, growth factors and morphogens are intrinsically unstable, while the ECM is both an active participant and is needed for dimensionality, as the examples in this review have shown. Thus, innovative biomaterial design and tissue engineering strategies are required involving: *i*) autologous or xenographic cells from the tissue to be formed; *ii*) signaling molecules which provide instruction for expressing a desire phenotype; and *iii*) synthetic scaffolds that hold the cells together and shape the tissue formation (Bason et al., 2018). Basically, the goal is to recapitulate the embryonic development and patterning process. Because of the prevalence of skeletal injuries, especially in the pediatric population 15–30% of pediatric skeletal injuries involve the growth plate (Shen et al., 2020), and because injury often results in replacement of cartilage by bone which precludes additional skeletal length growth (Gigante and Martinez, 2019), the growth plate is a highly desirable target for repair. However, in order to successfully re-engineer cartilage tissue, the natural characteristics of the growth plate, i.e., gradients of cell states, composition of ECM, position and function of growth regulators and mechanical properties must be replicated; the growth plate still remains an active model for studying tissue engineering strategies (Wang et al., 2021). Examples include: i) use of various cell types, i.e., bone marrow mesenchymal stem cells (BMSCs) or chondrocytes; ii) and various growth factors (TGFβ, IGF1, and FGF2) (Chen et al., 2020; Wei et al., 2020); iii) as well as different scaffolds composed of natural or synthetic material (Abdollahiyan et al., 2020), all with varying results. Mesenchymal stem cells (MSCs) are widely used in engineering of cartilage due to their capability for self-renewal and their ability to secrete multiple growth factors (Gultekin et al., 2020). As well, there is an influx of MSCs to the injured growth plate site, suggesting that MSCs are naturally vital to the repair process (Zhou et al., 2004). Several studies have shown that MSCs can be derived from multiple sources (Uder et al., 2018), with different regenerative potentials (Maheshwer et al., 2021). Other studies have used autologous chondrocytes which prevent bone formation, build the desired columnar structure and avoid immune rejection (Jin et al., 2006; Tomaszewski et al., 2014; Boopalan et al., 2019). Advances also continue to be made in developing three dimensional cultures that successfully retain the chondrogenic potential (Chow et al., 2011), as well culturing chondrocytes on synthetic hydrogels prior to seeding has been shown to lead to synthesis of Sox-9, aggrecan and collagen, which accumulate over time (Chang et al., 2018). As mentioned, manipulating the microenvironment by addition of chondrogenic-factors (TGFβ1, FGF-2, IGF-1, etc.) stimulates chondrogenesis and synthesis of ECM components (Thielen et al., 2019) (see previous sections). A major improvement in the regeneration process is the introduction of 3D printing technology, which allows different parts of the scaffold to have distinct porosity and mechanical properties, thus more faithfully recapitulating a natural cartilage growth plate (Shaw et al., 2018). In sum, to maintain viable cells, preserve growth factor stability, develop biocompatible as well as degradable natural (or synthetic) scaffolds is an extremely active research area in the field of growth plate regeneration summarized in Wang et al. (2021). Furthermore, many of the concepts are also shared with the bone regeneration field where appropriate combinations of scaffolding and seeding cells with growth factors are also being developed to engineer missing pieces of bone lost due to genetic malformations, trauma, tumors or infections (Perez et al., 2018; Koons et al., 2020; Alonzo et al., 2021). Although results continue to move in promising directions, there are still many challenges and unsolved problems that need to be resolved to benefit clinical application, as might be expected for recapitulating such a complex system such as the skeletal growth plate.

## Conclusion

Decades of studies have identified the hierarchical ECM-directed creation of the skeletal growth plate, which involves differentiation and growth of chondrocytes, positioning of cells, matrix and regulators into a highly integrated, bidirectional and temporally orchestrated process. As well, the reciprocity between ECM components, transcription factors, and signaling molecules that coordinate their expression has been revealed by the plethora of chondrodysplasias due to mutations in these regulatory molecules as well as proteoglycans and their biosynthetic enzymes that disrupt growth plate development and maturation summarized in Guasto and Cormier-Daire (2021). However, a more comprehensive molecular understanding is required to fully understand how chondrogenesis and growth plate expansion are regulated with such exquisite precision. As well, more detailed structural information to define the requisite structural interactions between CSPGs and pathway modulators are necessary to develop novel ECM/signaling paradigms, in order to reverse or ameliorate skeletal pathology.
